# The Role of Ayahuasca in Colorectal Adenocarcinoma Cell Survival, Proliferation and Oxidative Stress

**DOI:** 10.3390/ph17060719

**Published:** 2024-06-02

**Authors:** Joana Gonçalves, Mariana Feijó, Sílvia Socorro, Ângelo Luís, Eugenia Gallardo, Ana Paula Duarte

**Affiliations:** 1 Centro de Investigação em Ciências da Saúde (CICS-UBI), University of Beira Interior, 6200-506 Covilhã, Portugal; joanadgoncalves13@gmail.com (J.G.); marianapf95@gmail.com (M.F.); ssocorro@fcsaude.ubi.pt (S.S.); egallardo@fcsaude.ubi.pt (E.G.); apduarte@fcsaude.ubi.pt (A.P.D.); 2 Laboratório de Fármaco-Toxicologia, UBIMedical, University of Beira Interior, 6200-284 Covilhã, Portugal

**Keywords:** ayahuasca, Caco-2, viability, apoptosis, oxidative stress, cellular proliferation

## Abstract

The psychedelic beverage ayahuasca is originally obtained by *Banisteriopsis caapi* (*B. caapi*) (BC) and *Psychotria viridis* (*P. viridis*) (PV). However, sometimes these plant species are replaced by others that mimic the original effects, such as *Mimosa hostilis* (*M. hostilis*) (MH) and *Peganum harmala* (*P. harmala*) (PH). Its worldwide consumption and the number of studies on its potential therapeutic effects has increased. This study aimed to evaluate the anticancer properties of ayahuasca in human colorectal adenocarcinoma cells. Thus, the maximum inhibitory concentration (IC_50_) of decoctions of MH, PH, and a mixture of these (MHPH) was determined. The activities of caspases 3 and 9 were evaluated, and the cell proliferation index was determined through immunocytochemical analysis (Ki-67). Two fluorescent probes were used to evaluate the production of oxidative stress and the activity of the antioxidant enzymes superoxide dismutase (SOD) and glutathione peroxidase (GPx) was also evaluated. It was demonstrated that exposure to the extracts significantly induced apoptosis in Caco-2 cells, while decreasing cell proliferation. MH and MHPH samples significantly reduced oxidative stress and significantly increased glutathione peroxidase activity. No significant differences were found in SOD activity. Overall, it was demonstrated that the decoctions have a potential anticancer activity in Caco-2 cells.

## 1. Introduction

Ayahuasca is a hallucinogenic beverage from South America [1]. Traditionally, it was used by indigenous tribes in the Amazon for medicinal purposes and divine rituals [2]. The term ayahuasca is made up of the terms “*aya*” and “*wasca*” and means “vine of the dead” or “vine of the soul” [1,3]. This psychoactive beverage is obtained by boiling scrapings stem of *Banisteriopsis caapi* (Spruce ex Griseb.) C. V. Morton (*B. caapi*) (BC) and leaves of *Psychotria viridis* Ruiz & Pav. (*P. viridis*) (PV) resulting in a brownish, thick and oily drink [3]. Many iterations of this preparation have developed over time, and at this point, certain equivalents that can take the place of PV are known, (*Malouetia tamaquarina* A.DC., *Brugmansia suaveolens* (Willd.) Sweet, *Psychotria carthagenensis* Jacq., *Nicotiana tabacum* L., among others) as well as for BC (*Peganum harmala* L. (PH), tetrahydroharmine and harmine) [1,4].

The hallucinogenic character of ayahuasca is due to the presence of *N*,*N*-Dimethyltryptamine (DMT) from PV [5]. This compound is a serotonin receptor (5-HT1A/2A/2C) agonist, which when ingested alone is harmless, as it is metabolised by peripheral MAO-A [5]. However, this beverage also contains β-carboline alkaloids (harmine, tetrahydroharmine (THH) and harmaline) that come from BC [1,5]. This class of compounds is able to temporarily inhibit MAO-A, allowing DMT to access the bloodstream and subsequently the central nervous system [1]. Additionally, THH also inhibits serotonin reuptake enhancing the effects of DMT [6].

The rise in popularity of this psychoactive drink is also due to the fact that it is often seen as a natural remedy used for millennia to cure various ailments [7]. In spite of being used in traditional medicine, it is also consumed recreationally all over the world, representing a concern, since the laws that regulate this consumption are ambiguous and very variable [5,8].

Ayahuasca consumption is characterised by a set of physical symptoms, such as vomiting, nausea and diarrhoea, but also psychological effects such as changes in the perception of time and space, visual, auditory and cognitive effects [2,5]. However, several studies described the use of ayahuasca in the treatment of psychological problems such as depression, anxiety, addiction and psychological disorders [5]. Additionally, other studies have reported the ayahuasca antimicrobial, anti-inflammatory, and healing properties [9,10]. Furthermore, there is a great demand for ayahuasca as an alternative medicine for several diseases, including cancer, and its potential as a possible treatment for some types of cancer has been described [11].

Medical reports or clinical data about the potential of ayahuasca in treating cancer are almost non-existent [11]. However, isolated pieces of research work have suggested it could be interesting to be exploited in cancer therapy. A study available in the literature revealed that a liver cancer patient undergoing surgery to remove part of the organ achieved regression of carcinoembryonic antigens and remained in remission for five years after replacing the recommended chemotherapy with religious ayahuasca sessions [11]. Two other cases of prostate and ovarian cancer patients revealed significant improvements in the levels of prostate-specific antigen and CEA-125, respectively, after treatment with ayahuasca [12]. Favourable results from taking ayahuasca were also described in patients diagnosed with uterine [13] and breast [14] cancers. The present evidence, though scarce, strongly incites curiosity about investigating the anticancer role of ayahuasca and how it is propitiated from a mechanistic perspective.

Colorectal cancer is one of the main causes of death in the world, despite being studied for years [12]. In the previous decade, colon cancer accounted for 8.5% of all deaths worldwide and 9.7% of all cancer cases [13]. Sometimes, the cancer recurs, demonstrating that the available treatments are incomplete and may not be durable in the long term [14]. Therefore, finding new therapies or treatment adjuvants remains a scientific and clinical challenge. This study aimed to assess the impact of ayahuasca decoctions on the modulation of several characteristics of colorectal cancer cells, namely, cell viability, apoptosis, cell proliferation and oxidative stress.

## 2. Results and Discussion

In previous studies carried out by our research group, the main compounds present in the commercial mixture, also used here in the preparation of ayahuasca extracts, were determined [6,9]. After preparing the extracts, as described below, they were subjected to analysis by ultra-high performance liquid chromatography-quadrupole time-of-flight mass spectrometry (UHPLC/ESI-QTOF-MS) [9]. Briefly, after comparison with a phytochemical library of 48 standards, it was possible to quantify protocatechuic acid, 4-hydroxybenzoic acid, salicylic acid, (+)-catechin, (−)-epicatechin, quercetin-3-*O*-galactoside, quercetin-3-*O*-glucoside and quercetin-3-*O*-rutinoside [9]. In other studies, the main constituents of ayahuasca were characterised by high-performance liquid chromatography with diode-array detection (HPLC-DAD) [6] and gas chromatography coupled with mass spectrometry [15]. After preparing the commercial mixture, it was possible to quantify DMT, THH, harmine, harmaline and harmol [6]. However, it is important to highlight that in other extracts the concentrations of each compound in each ayahuasca sample can vary greatly, depending on the proportion used by each consumer, the preparation methods, the different concentrations of the compound between the plants, as well as its purity [6]. The toxicity of the extracts from the ayahuasca decoctions used herein was also evaluated in Caco-2 cell line, in another study carried out by our research group [6]. The results showed that the extracts did not present cytotoxicity.

### 2.1. Determination of the Half-Maximal Inhibitory Concentration (IC_50_)

The human colorectal adenocarcinoma Caco-2 cell line was chosen as our study model. In addition to the cancer-like phenotype, this cell line is widely used to mimic the intestinal mucosa barrier in absorption studies [16], gaining great pertinence in the present work as ayahuasca is consumed as a beverage. The IC50 values for all ayahuasca extracts in Caco-2 cells were determined by using the MTT assay and are shown in Table 1.

By evaluating the data, it was able to verify that the extract that presented a lower IC_50_ value, and therefore, requires a lower concentration to decrease the viability of Caco-2 cells, was the MHPH (IC_50_ = 276.97 μg/mL). The PH, commercial mixture, MHBC and PVPH extracts followed, with IC_50_ values in the range of 300 μg/mL, and the MH and BC extracts with IC_50_ values in the order of 500 μg/mL. The extracts with higher IC_50_ values were PVBC and PV with IC_50_ values of 623.54 μg/mL and 715.62 μg/mL, respectively. As far as we know, there are no studies determining the IC_50_ values of ayahuasca extracts in Caco-2 cells. Katchborian-Neto et al. [17] evaluated the cytotoxicity in cardioyocytes (CC_50_), these authors verified values greater than 200 μg/mL for DMT and ayahuasca (*P. viridis* and *B. Caapi*).

Considering the obtained results, the sample that presented the lowest IC_50_ value was MHPH, therefore it was selected to pursue the work. In the same way, the two samples that constitute the previous mixture (MH and PH) were also chosen to carry out the following assays, in order to understand the influence of each of these extracts on the results obtained for the MHPH sample.

### 2.2. Ayahuasca Extracts Affected Apoptosis in Caco-2 Cells

**Figure 1:**
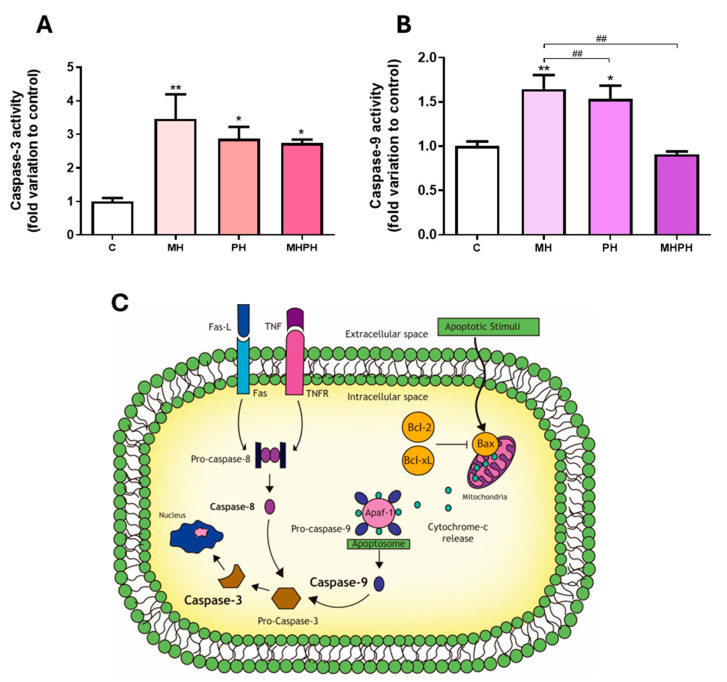
Caspase-3 (**A**) and caspase-9 (**B**) activity in Caco-2 cells behind the treatment with 276.97 µg/mL MH, PH and MHPH extracts for 24 h (n = 3). When compared to the control group, the error bars show the mean ± S.E.M. ANOVA *p* values: *p*  <  0.05 (*), *p*  <  0.01 (**), *p*  <  0.01 (##). (**C**) Intrinsic and extrinsic pathways of apoptosis. Two different mechanisms can cause apoptosis: the receptor-mediated (extrinsic) and the mitochondrial (intrinsic). The death receptors triggering the extrinsic pathway (e.g., Fas and tumour necrosis factor receptor, TNFR) are located at the plasma membrane and activated by their ligands (Fas-L and TNF, respectively), prompting the activation of the initiator caspase-8. The intrinsic route is triggered by several apoptotic stimuli that increase the ratio of proapoptotic (e.g., Bax)/anti-apoptotic (e.g., Bcl-2, Bcl-xL) mitochondrial proteins, causing the mitochondria to release cytochrome c. In the cytoplasm, cytochrome-c, pro-caspase-9 and the protease activating factor (Apaf-1) form the apoptosome, activating the initiator caspase-9. Pro-caspase-3 is where both routes converge and, after cleavage, becomes the active effector caspase-3, determining the end and an irreversible point of apoptosis.

Apoptotic programmed cell death is a critical biological process in maintaining tissue homeostasis and serves as a natural barrier to cancer development. Thus, impaired apoptotic responses significantly contribute to tumour progression and treatment resistance [18]. Herein, apoptosis was determined by measuring the activity of the executioner caspase-3, a target player in apoptotic cell death at the crossroads of the intrinsic and extrinsic apoptotic pathways [19] (Figure 1). Observing the results presented in Figure 1, it was possible to see that, in general, ayahuasca extracts increased caspase-3 activity in Caco-2 cell line. After exposing cells to PH and MHPH extracts, caspase-3 activity increased significantly, in comparison to the control group (2.87 ± 0.35 and 2.73 ± 0.12-fold increase, respectively, *p* < 0.05, Figure 1A). Regarding the MH extract, the results showed a more pronounced effect increasing caspase-3 activity (3.46 ± 0.73-fold increase, *p* < 0.01, Figure 1A).

In many cell line models, measuring caspase-3 activity has been utilised as a means of determining the rate of apoptosis [19,20,21]. Therefore, the results obtained clearly demonstrate the ability of ayahuasca extracts to induce apoptosis in Caco-2 cells, which is in accordance with the existing literature. In a study developed by Shabani et al. [22], where the triggering of apoptosis in the MDA-MB-231 breast cancer cell line was evaluated by PH extract, an increase in the induction of apoptosis through the intrinsic pathway was observed. Another study in breast cancer cell lines (MDA-MB-231 and MCF-7) also demonstrated an increase in apoptosis induced by harmine present in PH [23]. Li et al. [24] evaluated the induction of apoptosis in human gastric cancer cells, caused by harmine present in PH, and found an increase in the induction of apoptosis. Similar results were verified by Zhang et al. [25] and by Wang et al. [26]. Similarly, it was found that the same compound induced apoptosis in B16F-10 melanoma cells [27], in human colorectal carcinoma SW620 cells [28] and in non-small cell lung cancer (NSCLC) cells [29]. Otherwise, harmaline, present in PH, was responsible for arresting the cell cycle and inducing apoptosis in the glioblastoma cell line [30]. Other studies were carried out, where only the anticancer effects of synthetic or isolated β-carbolines were evaluated. It was found that harmine, harmaline, harmol or harmalol were able to increase the rate of apoptosis in human liver carcinoma cells [31,32], two lung tumour cell lines [33] and kidney adenocarcinoma cells [34].

As far as we know, no research was conducted to assess how MH extracts affect cancer cell apoptosis. On the other hand, it is possible to verify that this extract causes a significant increase in caspase-3 activity based on the results shown in Figure 1A. Thus, we can infer that the MH extract will be the most promising extract in inducing apoptosis in Caco-2 cells.

In order to understand which of the apoptotic pathways are being activated (intrinsic or extrinsic) in response to ayahuasca extracts treatment, the activity of caspase-9 was determined. This is the initiator caspase associated with the activation of the intrinsic (mitochondrial) pathway of apoptosis (Figure 1C) [35]. Currently, the intrinsic apoptotic pathway is widely implicated as a barrier to the carcinogenic process [18]. Observing the results presented in Figure 1B, it was possible to verify that there was a significant increase in Caco-2 cells’ caspase-9 activity, following exposure to PH and MH extracts (*p* < 0.05 and *p* < 0.01, respectively, Figure 1B). Thus, the results obtained suggest that apoptosis in cells exposed to PH and MH extracts occurred due to activation of the intrinsic pathway. These results are in accordance with the available scientific literature, showing that PH extracts are capable of inducing apoptosis in cancer cells, by activating the intrinsic pathway [22,28,36]. In a study developed by Elansary et al. [36], the activation capacity of caspase-9 was evaluated in cancer cells treated with PH extract compared to the control. An increase in caspase-9 activity was found in T lymphocyte lineage (Jurkat), bladder cancer (T24), colorectal adenocarcinoma (HT-29), breast cancer (MCF-7) and HeLa cells [36]. Another study, developed by Liu et al. [28], found that harmine, present in PH extracts, was also capable of activating caspase-9 in SW620 cells. Otherwise, no studies were found evaluating the effect of MH on caspase-9 activity.

Exposing Caco-2 cells to the MHPH extract, despite increasing the activity of caspase-3, did not alter caspase-9 activity, indicating that the apoptotic process may also be driven by the activation of the extrinsic pathway (Figure 1C). To our knowledge, no studies were performed evaluating the effect of the MHPH mixture on caspase-9 activation in Caco-2 cells, and further research is needed to disclose the involvement of membrane cell death receptors (extrinsic pathway) triggering apoptosis.

### 2.3. Ayahuasca Extracts Affected Cellular Proliferation in Caco-2 Cells

**Figure 2:**
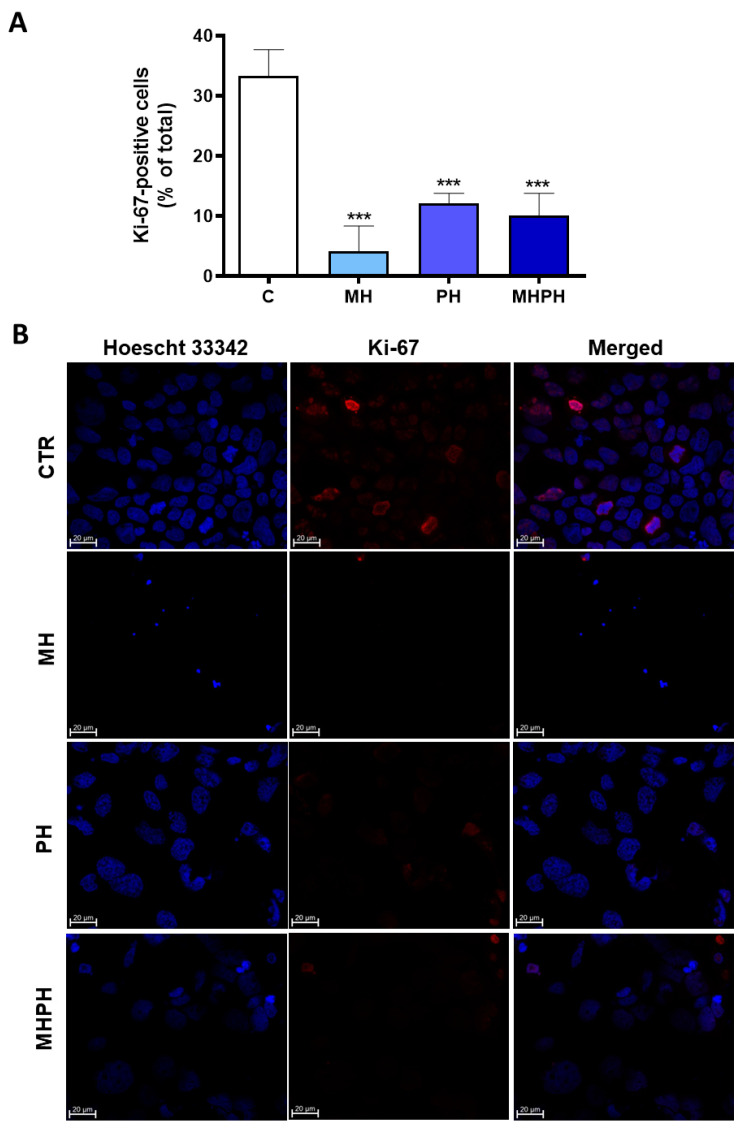
Proliferation index of Caco-2 cells after treatment with 276.97 µg/mL MH, PH and MHPH extracts for 24 h determined by Ki-67 immunofluorescence analysis (n = 3). (**A**) Proportion of cells that are positive for Ki-67 compared to all cells. The fold variation between the treated and untreated control groups is used to express the results. Mean ± S.E.M. is shown by error bars; ANOVA *p* values: *** *p* < 0.001. (**B**) Confocal microscopy photos of the control and treated groups that are representative of the Ki-67 labelling (red). Images were captured at a magnification of 630× using the Zeiss LSM 710 laser scanning confocal microscope (Carl Zeiss, Gottingen, Germany). Hoechst 33342 is used to stain the nuclei (blue).

Uncontrolled cell proliferation is one of the most recognised hallmarks of cancer [18]. Through Ki-67 immunofluorescence investigations, the proliferation index of Caco-2 cells treated with ayahuasca extracts was determined. It was established the number of Ki-67 positive cells there were in relation to all cells (Figure 2).

Ki-67 is widely used as a cell proliferation marker, as it is found in the nuclei of cells in a proliferative process at any stage of the cell division cycle [37]. Contrariwise, it is absent in cells that are not proliferating [37]. Ki-67 proliferation index of Caco-2 cells was significantly reduced in all treated groups compared to the control (Figure 2A). It was possible to verify that cells treated with the MH extract showed a greater reduction in the number of Ki-67 labelled cells (Figure 2A,B) and, therefore, a greater reduction in cell proliferation index (*p* < 0.001). The cell proliferation index of treated cells with MHPH and PH extracts was significantly lower than that of untreated cells (*p* < 0.001 for both groups). So far, there are no studies evaluating the influence of MH extracts on proliferation of cancer cells; however, some studies with PH extracts were developed. Wang et al. [38] verified that PH demonstrated antiproliferative effects in human lung cancer cells (A549). Other studies have shown similar results in gastric cancer cells [25,26], breast cancer cell lines (MDA-MB-231 and MCF-7) [23], oesophageal squamous cell carcinoma [39], NSCLC [29,40], glioblastoma [30], carcinoma (Med-mek and UCP-Med) and sarcoma (UCP-Med and Sp2/O-Ag14) [41,42] and human colorectal carcinoma (SW620) [28].

### 2.4. Ayahuasca Extracts Affected Oxidative Damage and Activity of Antioxidant Enzymes in Caco-2 Cells

**Figure 3:**
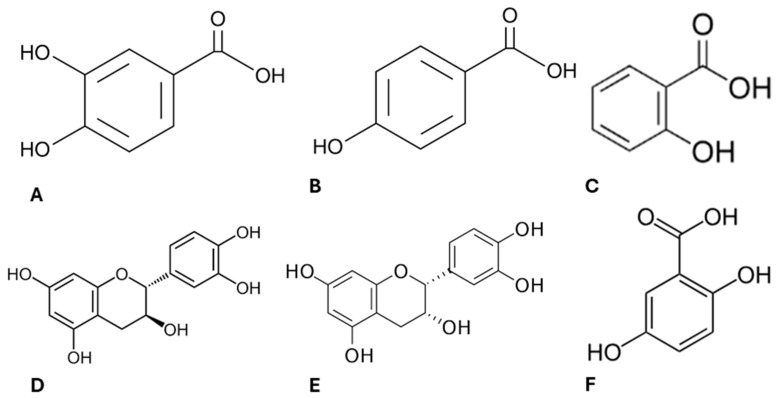
Chemical structure of the active compounds present in ayahuasca extracts. Protocatechuic acid (**A**), 4-hydroxybenzoic acid (**B**), salicylic acid (**C**), (+)-catechin (**D**), (−)-epicatechin (**E**), gentilic acid (**F**).

Lipids are the main macromolecules in the constitution of cell membranes and are highly sensitive to oxidative stress [35]. Damage to the structure and function of the lipid bilayer can compromise cell integrity [43], and this is one of the reasons why reactive oxygen species (ROS) have been considered in cancer therapy [44]. On the contrary, chronic increased levels of ROS are associated with the onset and development of cancer [45]. Therefore, as we have previously characterised the phytochemical profile of ayahuasca extracts [9] confirming its composition enriched in several compounds with antioxidant properties (Figure 3), we decided to investigated their effect in modulating oxidative stress in Caco-2 cells.

**Figure 4:**
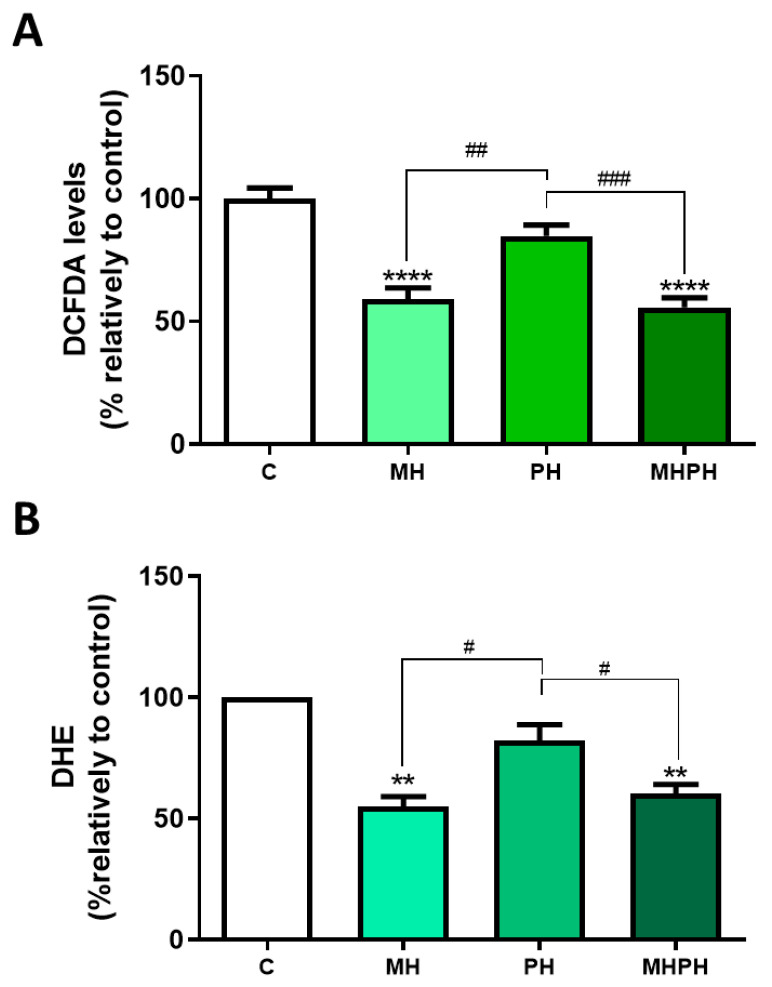
Cellular ROS production in Caco-2 cells after treatment with 276.97 µg/mL MH, PH and MHPH extracts for 24 h measured using dichlorofluorescein diacetate (DCFDA, (**A**)) or dihydroethidium (DHE, (**B**)) (n = 3). The fold variation between the treated and untreated control groups is used to express the results. The mean ± S.E.M., ANOVA *p* values: *p* < 0.05 (#), *p* < 0.01 (##), *p* < 0.001 (###), *p* < 0.01 (**), and *p* < 0.0001 (****) are indicated by the error bars.

In this study, ROS levels were determined by two different methods, with concordant results obtained. Results presented in Figure 4 show that the MH and MHPH extracts significantly reduced oxidative stress, as indicated by the diminished ROS levels (DCFDA, both *p* < 0.0001; DHE, *p* < 0.0001 and *p* < 0.001, respectively). On the other hand, no significant changes were observed with the PH extract. So far, there are no studies evaluating ROS levels in cancer cells after treatment with ayahuasca. However, there are several studies where the phytochemical profile of these extracts was evaluated, being found that compounds with antioxidant properties are part of their composition, which help to combat oxidative damage [9,36]. At first sight, these results appear controversial as a reduction in ROS levels may be considered a cell survival stimulus rather than an anti-tumoural one. However, it is important to enforce that chronic excessive cellular oxidative stress is widely perceived as a key factor in cancer development [46,47]. Thus, the ability of ayahuasca extracts to reduce ROS levels may represent another possible anti-cancer mechanism that deserves further investigation.

**Figure 5:**
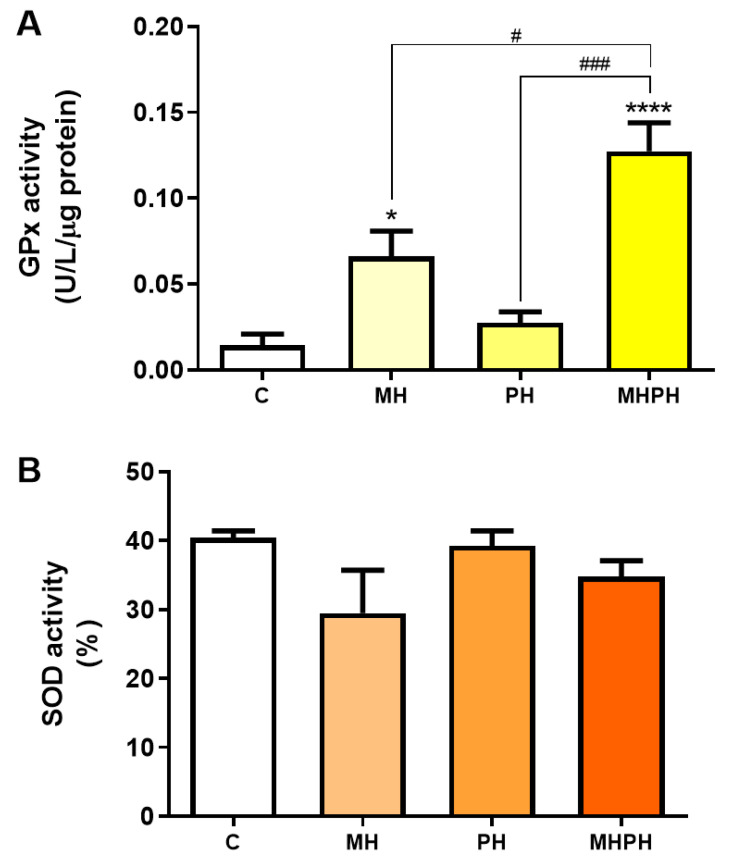
Activity of glutathione peroxidase (GPx, (**A**)) and superoxide dismutase (SOD, (**B**)) in Caco-2 cells behind the treatment with 276.97 µg/mL MH, PH and MHPH extracts for 24 h (n = 3). Enzyme activity is normalised to protein content. When compared to the control group, the error bars show the mean ± S.E.M., ANOVA *p* values: *p* < 0.05 (#), *p* < 0.001 (###), *p* < 0.05 (*), and *p* < 0.0001 (****).

Another feature of cancer cells is the upregulated expression of antioxidant defence enzymes to maintain ROS levels within ranges that allow for avoiding cell death [48]. However, low levels of antioxidant enzymes have also been reported in certain types of cancer (e.g., bladder, cervical, breast), and mostly depending on the stage of disease [49,50,51]. GPx and SOD enzymes play an important role in the defence against oxidative stress [35]. When evaluating GPx activity (Figure 5A) there was a significant increase after exposure to the MH and MHPH extracts (*p* < 0.05 and *p* < 0.0001, respectively) and a non-significant increase in the PH sample. However, no significant differences were found in the evaluation of SOD activity (Figure 5B). These results indicated that the tested ayahuasca extracts have a good potential for minimising oxidative damage in Caco-2 cells by increasing the activity of the GPx enzyme, though not affecting the SOD activity. In fact, Bourogaa et al. [52] evaluated the protective effects of the PH extract in the chronic treatment with ethanol. After treatment with PH extract, GPx activity increased [52]. The same results were verified for SOD activity [52]. These results can be justified by the antioxidant activity of the PH extract, which may be involved in an inhibition effect on damage caused by ROS, leading to an increase in endogenous antioxidant activity [52]. In addition to the antioxidant activity attributed to the PH extract, it may also be involved in the scavenging of free radicals and inhibition of lipid peroxidation [52]. However, so far, no research has been conducted with MH samples, evaluating the activity of antioxidant enzymes.

Ayahuasca decoctions (MH, PH and MHPH) were able to induce apoptosis and reduce the viability and proliferative activity of Caco-2 cells. Additionally, it was demonstrated that oxidative stress levels decreased in the presence of extracts, and despite no significant differences being detected in SOD activity, the results of GPx activity suggest that the extracts can trigger a defence response against oxidative stress. A study previously carried out by our research group evaluated the phytochemical profile of these ayahuasca extracts [9]. It was found that several compounds with antioxidant properties, such as protocatechuic acid, 4-hydroxybenzoic acid, salicylic acid, (+)-catechin [53], (−)-epicatechin [54], and gentisic acid [55] are part of its composition. Therefore, it is likely to assume that these compounds may be involved in combating oxidative damage [9,36] justifying the results obtained herein.

## 3. Materials and Methods

### 3.1. Plant Material and Preparation of Extracts

On 25 May 2019, we obtained vegetable samples via the internet from Shayana Shop (https://www.shayanashop.com, Amsterdam, The Netherlands) (accessed 25 May 2019). “World Flora Online” (www.worldfloraonline.org accessed 15 December 2023) was used to verify the full names of botanical plants. The five vegetal samples (commercial mixture, PV leaves, BC stem scraps, PH seeds, and MH root bark) were weighed in order to prepare ayahuasca decoctions. Following that, the plant material was milled in a mortar with a few drops of water before being moved and mixed with 250 mL of ultrapure water in a Schott flask. The mixture was then brought to a boil for 4 h at 100 °C. Similarly, four decoctions were prepared by mixing two of the selected plants (PV and PH (PVPH); PV and BC (PVBC); MH and PH (MHPH) MH and BC (MHBC)). Following filtration, the samples were frozen at 80 °C and then placed in the freeze dryer until they were completely freeze-dried.

### 3.2. Cell Culture and Treatment

Caco-2 cells were purchased from the American Type Culture Collection (ATCC) (Accession number: HTB-37) and maintained in Roswell Park Memorial Institute (RPMI) 1640 culture medium (Sigma-Aldrich, Sintra, Portugal), supplemented with 10% foetal bovine serum (FBS) and 1% antibiotic mixture (Sigma-Aldrich), at 37 °C in an air incubator with a humidified atmosphere of 5% CO_2_. For analysis of the effects of ayahuasca extracts on cell viability, Caco-2 cells were cultured in 96-well plates (cat. number 734-2802 Avantor, VWR, Amadora Portugal) with 1, 50, 250, 500, 750 and 1000 μg/mL of extract, prepared in culture medium, for 24 h. Apoptosis, cell proliferation (96-well plates) and oxidative stress (96-well plates) were assessed using extracts at 276.97 μg/mL (concentration corresponding to the IC_50_ of the MHPH sample) for a treatment time of 24 h. For all assays RPMI medium was used as a negative control.

### 3.3. Cell Viability Assay

The MTT assay was used to analyse cell viability. This method consists in the reduction of 3-(4,5-dimethylthiazol-2-yl)-2,5-diphenyltetrazolium bromide into its insoluble formazan (Sigma-Aldrich). Cells were exposed to MTT until formazan crystals were obtained (3 to 4 h), which were thereafter dissolved with 200 μL of dimethyl sulfoxide (DMSO). The absorbance at 570 nm was measured using the xMark^TM^ microplate absorption spectrophotometer (Bio-Rad Laboratories, Hercules, CA, USA). All experiments were performed in three independent assays [6].

### 3.4. Protein Extraction

By homogenising the Caco-2 cells in the appropriate volume of radioimmunoprecipitation (RIPA) buffer—150 mM NaCl, 1% Nonidet-P40 substitute, 0.5% Nadeoxycholate, 0.1% SDS, 50 mM Tris pH 8.0, and 1 mM EDTA—supplemented with 10% PMSF and 1% protease inhibitor cocktail (Sigma-Aldrich), the total protein was extracted from the cells. The homogenates of cells were centrifuged at 14,000 rpm for 20 min at 4 °C after being on ice for 20 min and shaken periodically. Using the bicinchoninic acid (BCA) test (Thermo Fisher Scientific, Rockford, IL, USA), the total protein content of the supernatant was determined [35].

### 3.5. Caspase-3 and Caspase-9 Activity Assays

The activity of Caspase-3 and Caspase-9 was determined using the Caspase-3 Assay Kit (Sigma-Aldrich) and Caspase-9 Colorimetric Assay Kit (Sigma-Aldrich), respectively. By quantifying the release of the p-nitroaniline chromophore group (pNA) through cleavage of their respective substrates (Ac-DEVD-pNA and LEHD-pNA, respectively), the activities were ascertained spectrophotometrically. Consequently, an appropriate volume of reaction buffer (25 mM HEPES, pH 7.5, 0.1% 3-[(3-cholamidopropyl) dimethylammonium]-1-propanesulfonate, 10% sucrose, and 10 mM dithiothreitol (DTT) containing 200 mM of substrate) was incubated with 3 μL of total protein extracts at 37 °C overnight. An xMark^TM^ microplate absorption spectrophotometer (Bio-Rad Laboratories, Hercules, CA, USA) was used to measure the release of pNA at 405 nm. Caspases 3 and 9 activity was estimated by extrapolating the amount of released pNA using a standard free pNA curve [35].

### 3.6. Ki-67 Fluorescent Immunocytochemistry

After 10 min of paraformaldehyde (4%) fixation, Caco-2 cells were permeabilised for 5 min using Triton (1%) solution. Following this, cells were incubated for 1 h at room temperature in phosphate buffer saline (PBS) with 0.1% Tween 20^®^ (PBST) and 20% FBS as a blocking phase. Following a washing step, the cells were treated for 1 h at room temperature with the primary rabbit anti-Ki-67 antibody (1:50, no. 16667, Abcam, Cambridge, UK). Following that, cells were treated for another hour at room temperature with Alexa fluor 546 goat anti-rabbit IgG secondary antibody (1:500, Invitrogen, Paisley, Scotland). The specificity of the immunostaining was evaluated by excluding the primary antibody and staining the cell nuclei for 10 min with Hoechst 33342 (5 μg/mL, Invitrogen). Following a wash, Dako fluorescent mounting media (Dako, Glostrup, Denmark) was used to fix the coverslips on the slide. Images were acquired using a Zeiss LSM 710 confocal laser scanning microscope (Carl Zeiss, Göttingen, Germany), and the proliferation index was estimated by counting the number of Ki-67-positive cells and Hoechst-stained nuclei in 10 randomly selected fields for each section at 63× magnification. The ratio between the number of Ki-67-stained cells and the total number of nuclei was calculated [35].

### 3.7. Cellular Reactive Oxygen Species (ROS) Level Measurements

The ROS level was determined using two fluorescent probes: DCFDA (Sigma-Aldrich) and DHE, which measures cytosolic superoxide (Sigma-Aldrich). DCFDA is oxidised by ROS and transformed into fluorescent 2′,7′-dichlorofluorescein, which is the basis for the DCFDA assay. Following 24 h exposure to ayahuasca extracts, culture medium was removed, and cells were treated for 1 h at 37 °C with 50 μM DCFDA prepared in the culture medium. The emitted fluorescence was read in a spectrofluorometer (SpetroMax Gemini EM; Molecular Devices, San José, CA, USA) at 485 (excitation) and 535 nm (emission) [56]. On the other hand, red fluorescent ethidium bromide is created when superoxide (O_2_^−^) dehydrogenates blue fluorescent DHE. Likewise, cells were treated with 100 μM DHE in culture media at 37 °C for 20 min after being exposed to the stimuli for 24 h. A spectrofluorometer (SpetroMax Gemini EM) was used to measure the produced fluorescence (excitation 515 nm; emission 605 nm) [57].

### 3.8. Glutathione Peroxidase Assay

Using a commercial kit (Calbiochem, Darmstadt, Germany), GPx activity was assessed in accordance with the manufacturer’s instructions. The method focuses on the oxidation of glutathione (GSH) to oxidised glutathione (GSSG), which occurs at a temperature of 25 °C and is catalysed by GPx. The actions of glutathione reductase (GR) and NADPH convert GSSG back into GSH. The absorbance at 340 nm (xMarkTM Microplate Absorbance Spectrophotometer (Bio-Rad)), which is proportional to GPx activity, decreases when NADPH is oxidised to NADP+ [35].

### 3.9. Superoxide Dismutase Assay

The commercial SOD Assay Kit-WST (Sigma-Aldrich) was used to measure SOD activity in accordance with the manufacturer’s instructions. Briefly, the reduction of the WST-1 substrate (tetrazolium salt) with a superoxide anion results in the production of a water-soluble formazan colour. The activity of xanthine oxidase (inhibited by SOD) is linearly related to the rate of formazan production. The xMark^TM^ Microplate Absorbance Spectrophotometer (Bio-Rad) was used to measure the formazan production at 450 nm in relation to the amount of superoxide anion and the reduction reaction at 37 °C. The percent inhibition rate of the reaction corresponds to the SOD activity [35].

### 3.10. Statistical Analysis

The statistical analyses were all carried out with GraphPad Prism8. Tukey’s test was conducted after the results of the Student’s *t* test or ANOVA to determine the statistical significance of the various groups. If *p* < 0.05 (*), *p* < 0.01 (**), *p* < 0.001 (***), or *p* < 0.0001 (****), there were significant differences. Every experimental result is displayed as mean ±  S.E.M.

## 4. Conclusions

Ayahuasca decoctions were able to induce apoptosis and reduce the viability and proliferative activity of Caco-2 cells. Oxidative stress levels decreased in the presence of ayahuasca extracts and, although no significant differences were observed in SOD activity, the results of GPx activity suggest that extracts can trigger a defence response against oxidative damage. The current findings have added to our understanding of the biological effects of ayahuasca extracts, demonstrating the anticancer properties of this natural product. Moreover, this study opens new research lines to further explore the potential of ayahuasca extracts as anticancer agents by in-depth studies, namely in vivo assays and clinical trials. Future studies should fully address the molecular mechanisms through which ayahuasca extracts exert their tumour suppressor effects by evaluating tumour-specific markers as precursors of disease. Moreover, the anticancer potential of ayahuasca over other cell lines should be envisaged.

## Figures and Tables

**Table 1:** IC_50_ values of ayahuasca extracts in Caco-2 cells after treatment for 24 h.

Sample	IC_50_ (μg/mL) ± SD
PV	715.62 ± 0.05
BC	558.42 ± 0.08
PH	338.99 ± 0.05
MH	553.58 ± 0.02
Commercial Mixture	365.42 ± 0.02
PVBC	623.54 ± 0.07
PVPH	375.97 ± 0.02
MHBC	366.17 ± 0.05
MHPH	276.97 ± 0.05

PVBC (*P. viridis* and *B. caapi*); PVPH (*P. viridis* and *P. harmala*); MHBC (*M. hostilis* and *B. caapi*); MHPH (*M. hostilis* and *P. harmala*).

## Data Availability

The data are all available in the article.
